# Theoretical Prediction of 2D Y_2_CTI (T = Br, Cl, F, H) Janus MXene Monolayers for Photovoltaic Applications

**DOI:** 10.1021/acsomega.5c05916

**Published:** 2025-09-24

**Authors:** Bill D. Aparicio-Huacarpuma, Calos M. de Oliveira Bastos, José A. S. Laranjeira, Fábio L. L. Mendonça, Alysson M. Almeida Silva, Julio R. Sambrano, Alexandre Cavalheiro Dias, Luiz Antônio Ribeiro Júnior

**Affiliations:** † Institute of Physics, 564113 University of Brasília, Brasília 70919-970, DF, Brazil; ‡ Computational Materials Laboratory, LCCMat, Institute of Physics, 564113 University of Brasília, 70910-900 Brasília, Brazil; ¶ Institute of Physics and International Center of Physics, 564113 University of Brasília, Brasília 70919-970, DF, Brazil; § Modeling and Molecular Simulation Group, School of Sciences, 28108 São Paulo State University (UNESP), Bauru 17033-360, SP, Brazil; ∥ College of Technology, Department of Electrical Engineering, University of Brasília, 70910-900 Brasília, Brazil; ⊥ College of Technology, Department of Mechanical Engineering, University of Brasília, 70910-900 Brasília, Federal District, Brazil

## Abstract

Two-dimensional Janus monolayers have garnered attention as promising materials for photovoltaic applications due to their distinctive structural, electronic, and optical properties. In this work, we investigate the solar harvesting efficiency of Janus Y_2_CTI (T = Br, Cl, F, H) MXene monolayers using density functional theory and a maximally localized Wannier function tight-binding framework. Our results demonstrate an outstanding power conversion efficiency ranging from 31 to 32% for the investigated materials. These values were calculated at 300 K, incorporating the quasi-particle effects and considering the constraints imposed by the Shockley–Queisser limit. Furthermore, excitonic effects induced by quantum confinement contribute to exciton binding energies between 228 and 325 meV and indirect band gaps ranging from 1.23 to 1.33 eV, depending on compounds. Our results indicate a high potential of Janus Y_2_CTI MXene monolayers for photovoltaic applications and provide insights into their excitonic contribution.

## Graphical abstract

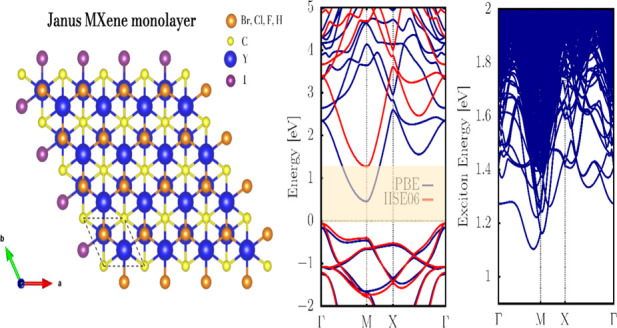

## 1 Introduction

Since the discovery of graphene,^1^ two-dimensional (2D) materials have been extensively explored in both experimental and theoretical research owing to their remarkable chemical and physical properties. Among the most studied 2D materials are transition metal dichalcogenides (TMDs),^2–4^ boron nitride,^5^ phosphorene,^6^ silicene,^7^ germanene,^8,9^ carbon allotropes,^10^ and MXenes.^11^

Different photovoltaic materials were researched recently for photovoltaic systems^12^ and solar-blind deep UV photodetectors,^13,14^ and the fabrication of these materials is challenging in PV systems. Also, flash memory uses 2D materials as a storage with low voltage, showing the importance of 2D materials for these types of applications.^15^ To enhance photovoltaic power generation in desert environments, CO_2_-based binary mixtures were analyzed in a supercritical Brayton cycle. CO_2_-propane showed the highest efficiency gains, improving thermal and energy performance under high ambient temperatures.^16^ Also, Ti_3_C_2_T_*x*_ 2D MXene materials show potential for advanced photovoltaic and energy harvesting systems experimentally.^17–19^

In particular, over the past decade, MXenes have received interest in electronics due to their exceptional physical and electronic properties.^20^ Since the pioneering synthesis of MXenes by Naguib et al. in 2011,^21^ these materials have gained attention in theoretical and experimental studies due to their interesting properties. MXenes are produced through the exfoliation method of the MAX Ti_3_AlC_2_ phase,^21,22^ where M corresponds to an early transition metal,^11,23^ A is an element from groups 13 (IIIA) or 14 (IVA) on the periodic table of chemical elements,^24^ and X indicates carbon (C) or nitrogen (N). These systems are generally expressed as M_*n*+1_X_*n*_ and can be functionalized with a surface from groups 16 and 17 of the periodic table. Their extended formula is M_*n*+1_X_*n*_T_*x*_ (*n* = 1, 2, 3, or 4),^25,26^ where T_*x*_ represents surface terminations.

The surfaces of MXenes can be independently or simultaneously functionalized, enabling dual-surface modification, as observed in structures such as M_2_CTT′,^27^ or in carbide frameworks involving two different metals, such as MM′CT_2_.^28^ MXene materials have demonstrated significant potential across a wide range of applications, including energy storage,^29,30^ transparent materials,^31^ sensors and gas adsorption,^32,33^ catalysis,^34^ photovoltaics,^35^ and spintronic devices.^36^

Kumar et al. explored the excitonic, electronic, and optical properties of semiconducting hafnium-based MXenes, and the results indicated the promising potential of these materials in optoelectronic devices.^37^ Recently, Aparicio-Huacarpuma et al. investigated the electrical and optical properties of 2D MXene monolayers based on scandium and yttrium with different surface terminations, showing the potential for solar harvesting efficiency and the contribution of the excitonic effect induced by quantum confinement.^35^ However, there is no research on Janus MXenes with different T_*x*_ terminations for potential applications in photovoltaic devices, and this remains an open question.

The development of Janus 2D materials, by introducing asymmetry at the atomic scale, has expanded the potential for atomically engineered material design. For example, Janus Sc_2_C-based MXene monolayers exhibit unique properties from their dual-surface nanometric structures, making these materials promising for diverse technological applications.^38,39^ Modi et al. studied the Y_2_CTT′ Janus monolayer, highlighting the electronic and optical properties of asymmetric systems functionalized with (Br, Cl, F).^27^ Also, in a previous work,^40^ the excitonic effect of Janus M_2_CTT′ (M = Y, Sc; T/T′ = Br, Cl, F) was explored with solar harvesting efficiency between 25.5 and 32.6%. Inspired by these Janus MXenes, we are proposing a new type of Janus MXene based on Y_2_CTT′.

In this work, we evaluate the structural, phonon spectra, and electronic properties, as well as the excitonic effects and optical properties, of the MXene monolayers derived from Y_2_CTT′ (T = Br, Cl, F, H and T′ = I). We used first-principles calculations based on the density functional theory (DFT) framework, complemented by a semiempirical method based on the maximally localized Wannier function tight-binding model (MLWF-TB)^41,42^ to examine these properties. Our results showed that these 2D Y_2_CTI monolayers exhibit optimal optoelectronic properties with good responses in the infrared (IR) and visible regions, making them strong candidates for photovoltaic and electronic applications due to the optimal band gap around 1.3 eV. Furthermore, the power conversion efficiency (PCE), estimated under the Shockley Queisser (SQ) limit^43^ and the spectroscopy limited maximum efficiency (SLME),^44^ indicates performance efficiencies ranging from 31% to 32%, establishing these materials as promising candidates for next-generation solar technologies.

## 2 Computational Details

In this study, we performed DFT^45,46^ calculations using the Vienna *Ab initio* Simulation Package (VASP version 6.4.3),^47,48^ employing the projector augmented wave (PAW) method.^49,50^ We utilized the Perdew–Burke–Ernzerhof (PBE) functional^51,52^ to evaluate the exchange and correlation (XC) terms for stress tensor relaxation, phonon, and electronic properties. A high kinetic cutoff energy of 800 eV was utilized to ensure numerical accuracy for the wave function. The Brillouin zone was sampled with dense **k**-point grids of 12 × 12 × 1 for the optimization process and 24 × 24 × 1 for the density of states and band structure calculations. A vacuum box of 25 Å was introduced perpendicular to the *xy* plane in order to eliminate spurious interactions between the periodic layer images of the Y_2_CTI monolayers. The convergence criterion based on the Hellman–Feynman theorem was set to 1 × 10^–6^ eV for the total energy and 0.01 eV/Å for the residual ionic forces.

To address the underestimation of the electronic band gaps inherent in the PBE functional with experimental and hybrid XC functionals,^53–56^ the Heyd Scuseria Ernzerhof (HSE06) hybrid functional^57^ was applied to obtain the best representation band structures and correct the electronic band-gap value. Structural visualizations and analyses were performed using VESTA software,^58^ and the VASPKIT toolkit facilitated data postprocessing.^59^ Phonon dispersion curves, computed using Phonopy, were used to assess the dynamical stability^60^ interfaced with VASP (PBE functional), with a 2 × 2 × 1 supercell, maintaining the same **k**-points density employed in the optimization process.

*Ab initio* molecular dynamics (AIMD) simulations were carried out using the FHI-aims code,^61,62^ employing numerical atom-centered orbitals (NAOs) with the light first-tier basis set. All simulations were performed within the PBE XC-functional framework, using a 3 × 3 × 1 supercell with a 4 × 4 × 1 **k**-mesh. The calculations were conducted in the NVT ensemble, regulated by a Nosé–Hoover thermostat, at room temperature of 300 K. The total simulation time was 5 ps (5000 fs) with a time step of 1 fs.

Maximally localized Wannier functions (MLWFs) were also configured using the Wannier90 package^63^ to parametrize a tight-binding (TB) Hamiltonian derived from DFT calculations using the HSE06 hybrid functional. The excitonic effect and optical properties were calculated using the WanTiBEXOS code.^64^ Orbital projections included *d*-orbitals for Y, *p*-orbitals for C, Br, Cl, F, and I elements, and *s*-orbitals for the H element. Optical properties were obtained using the IPA method, which excludes excitonic effects, and at the BSE level, which accounts for quasi-particle interactions, with a **k**-points density of 120 Å^–1^. For the BSE calculations, we used a 2D-truncated Coulomb potential (V2DT)^65^ to consider the influence of reduced dimensionality on the system. We use a smearing of 0.05 eV for the dielectric functions. For the optical and excitonic properties, the three highest valence bands and lowest conduction band were considered, which is sufficient to describe the linear optical response in the solar emission spectrum range of 0.5–4.0 eV.^66^

The PCE of the Y_2_CTI monolayers was obtained using the WanTiBEXOS code, employing the SQ limit^43^ and the SLME approach^44^ at operating room temperature of 300 K. Unlike the SQ limit, which demands only the band gap (or exciton ground state if quasi-particle effects are considered), the SLME also considers the direct or indirect nature of the band gaps and the optical absorption coefficient employing the AM1.5G reference standard solar spectrum.^66,67^

The absorbance rate was calculated based on the total absorption coefficient and the system’s thickness, taking into account all diagonal components of the dielectric function. The monolayer thickness was specified as the intrinsic material thickness plus a van der Waals (vdW) length of 3.21 Å, as reported by Bernardi et al.^67^ This method has been shown to reliably estimate the absorbance of graphene and other 2D materials, yielding results that closely match the experimental results.

## 3 Results and Discussion

### 3.1 Structural, Stability, and Mechanical Properties

The perspective (left), side, and top views of Janus-like 2D Y_2_CTI (T = Br, Cl, F, H) MXenes are presented in Figure 1, highlighting their structural anisotropy due to asymmetric terminations. These monolayers have a trigonal layer group classification (*p*3̅*m*1) according to 2D lattice materials.^68^ In this particular atomic configuration, there are five layers, forming a T-M-C-M-I stacking (Y_2_CTI), with Y as the central metallic element. Carbon is represented by C, while T signifies the functional groups consisting of Br, Cl, another Cl, H, and an iodine (I) atom. From these structural arrangements, four unique Janus monolayer variations can be obtained, namely, Y_2_CBrI, Y_2_CClI, Y_2_CFI, and Y_2_CHI.

**Figure 1:**
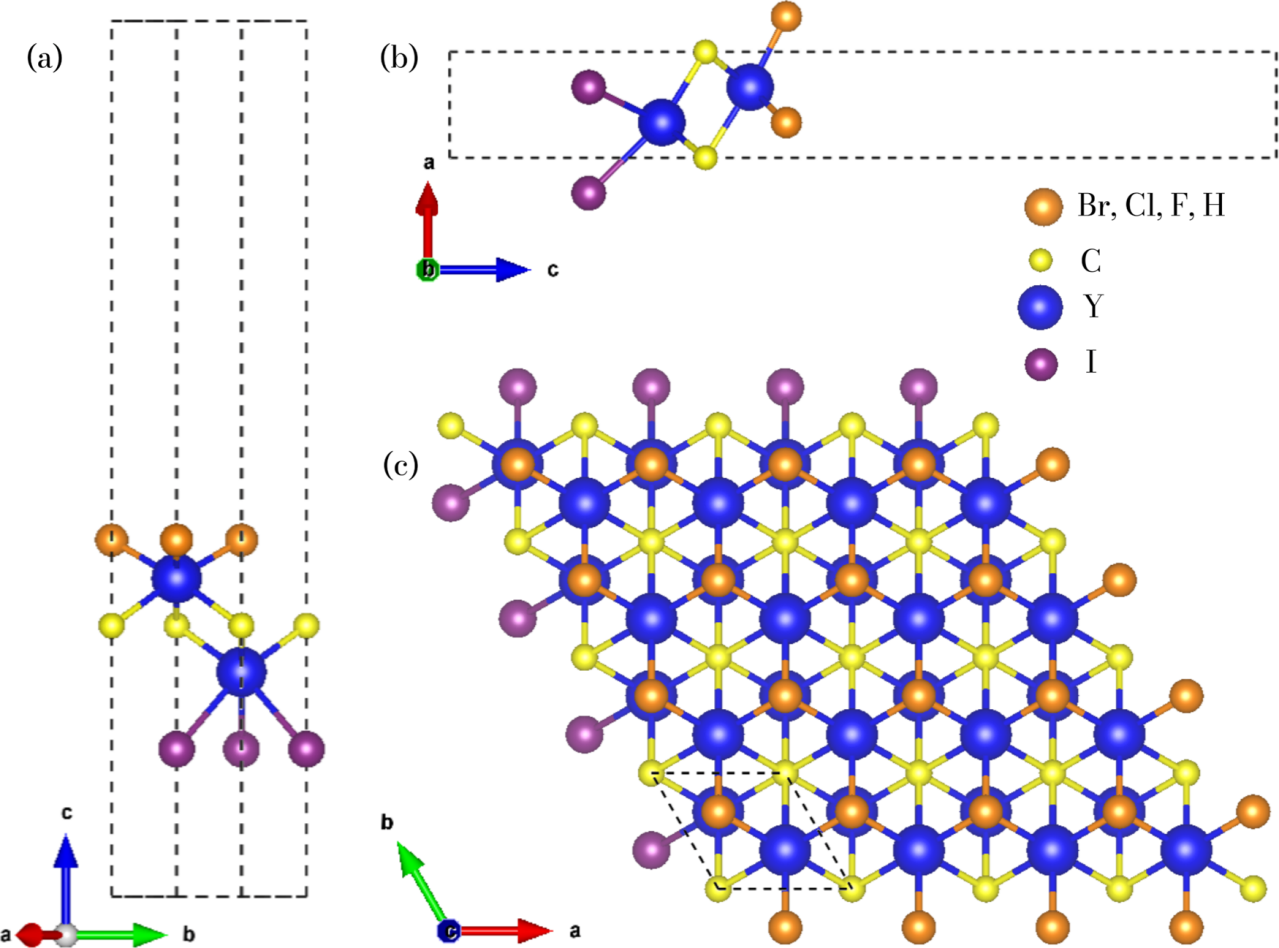
(a–c) Crystal structure views of trigonal 2D Y_2_CTI (T = Br, Cl, F, H) monolayers. The orange spheres represent the atoms Br, Cl, F, and H, and the yellow, blue, and violet spheres represent the atoms C, Y, and I, respectively. The black dashed line depicted in (c) represents the unit cell, and the dashed lines in (a) and (b) present a vacuum box of 25 Å for the Janus monolayers.

The lattice parameter *a*_0_ shows a slight decreasing trend from Y_2_CBrI (3.82 Å) to Y_2_CFI (3.72 Å), reflecting the decreasing atomic size of the T terminations. This contraction is consistent with the smaller covalent radius and stronger bonding character of fluorine. Concerning the monolayer thickness, including van der Waals length *t*_0_, a reduction is observed, as lighter atoms replace heavier halogens. Y_2_CBrI reaches the largest thickness (9.95 Å), while Y_2_CHI is the thinnest (9.10 Å), which is consistent with the steric effects and bond lengths associated with each terminating atom. Table 1 summarizes the cohesive energy (*E*_coh_), optimized lattice constant (*a*_0_), and thickness including van der Waals length (*t*_0_) for the studied 2D Y_2_CTI MXene monolayers.

**Table 1:** Calculated Cohesive Energy, *E*_coh_ (eV/atom), Calculated Heat of Formation, Δ*H* (eV/atom), Lattice Parameter (*a*_0_), and Monolayer Thickness with van der Waals Contribution (*t*_0_) for the Janus-like MXenes

system	*E*_coh_	Δ*H*	*a*_0_ (Å)	*t*_0_ (Å)
Y_2_CBrI	–4.947	–1.380	3.82	9.95
Y_2_CClI	–5.076	–1.475	3.80	9.77
Y_2_CFI	–5.401	–1.875	3.72	9.22
Y_2_CHI	–4.642	–0.873	3.74	9.10

For a theoretically predicted new material, it is crucial to assess its material formation as well as its dynamical, mechanical, and thermodynamic stabilities. First, to evaluate formation stability, we examine the cohesive energy (*E*_coh_), which can be obtained from the following expression:

$$E_{\mathrm{coh}}=\frac{E_{\mathrm{tot}}(M_{2}\mathrm{CTI})-\left(\sum_{i=M,C,T,I}N(i)E(i)\right)}{N(M_{2}\mathrm{CTI})}$$ 1

In these expressions, *E*_tot_M_2_CTI is referred to the minimal total energy of the pristine Janus MXene monolayer system, *E*(*i*) is the total energy of the isolated atom of Y, C, Br, Cl, F, H, and I, and *N*(*i*) denotes the number of atoms in the unit cell of each atom. For these systems, we have 2 atoms of Y and 1 atom for the others. The *N*(M_2_CTI) in the denominator is the total number of atoms; in our scenario, we have 5 atoms in the unit cell.

The cohesive energy being negative indicates that the attraction between the particles of the material is greater than the energy required to separate them, demonstrating the material’s stability. All investigated systems exhibit negative cohesive energies, indicating their stability in formation. Among them, Y_2_CFI shows the lowest *E*_coh_ value (−5.401 eV/atom), suggesting the highest stability, which stems from the high electron affinity and minimal atomic radius of F, in comparison with Br and Cl. In contrast, Y_2_CHI presents the least negative cohesive energy (−4.642 eV/atom).

To verify the thermodynamic stability, the heat of formation (Δ*H*), proposed in ref 69, was calculated. For this calculation, the most stable standard state of the elements involved was considered. The elements Y and C were considered to be solid. In the case of H and halogens such as F, Cl, Br, and I, their standard state is not metallic (they do not have an elemental crystal lattice), so they were considered diatomic molecules. Thus, the heat of formation equation, Δ*H*, was defined as

$$\Delta H=\frac{E_{\mathrm{tot}}(Y_{2}\mathrm{CTI})-E(2Y+C+T+I)}{5}$$ 2

where *E*_tot_(Y_2_CTI) is the total energy of the monolayer system and *E*(Y), *E*(C), *E*(T= H, F, Cl, Br), and *E*(I) are the total energies of the elements in their standard state. The four monolayer systems meet the Δ*H* < 0 requirement, making them thermodynamically stable. These values are listed in Table 1 and are in the range of −0.873 to −1.855 eV/atom.

To establish the stability of the systems, *ab initio* molecular dynamics (AIMD) was successfully run at room temperature (300 K), using the canonical NVT ensemble, for 5 ps. The energy fluctuations throughout the simulations are depicted in Figure 2. The energy remained relatively stable during the considered period, indicating the absence of a reconstruction or bond breakage events.

**Figure 2:**
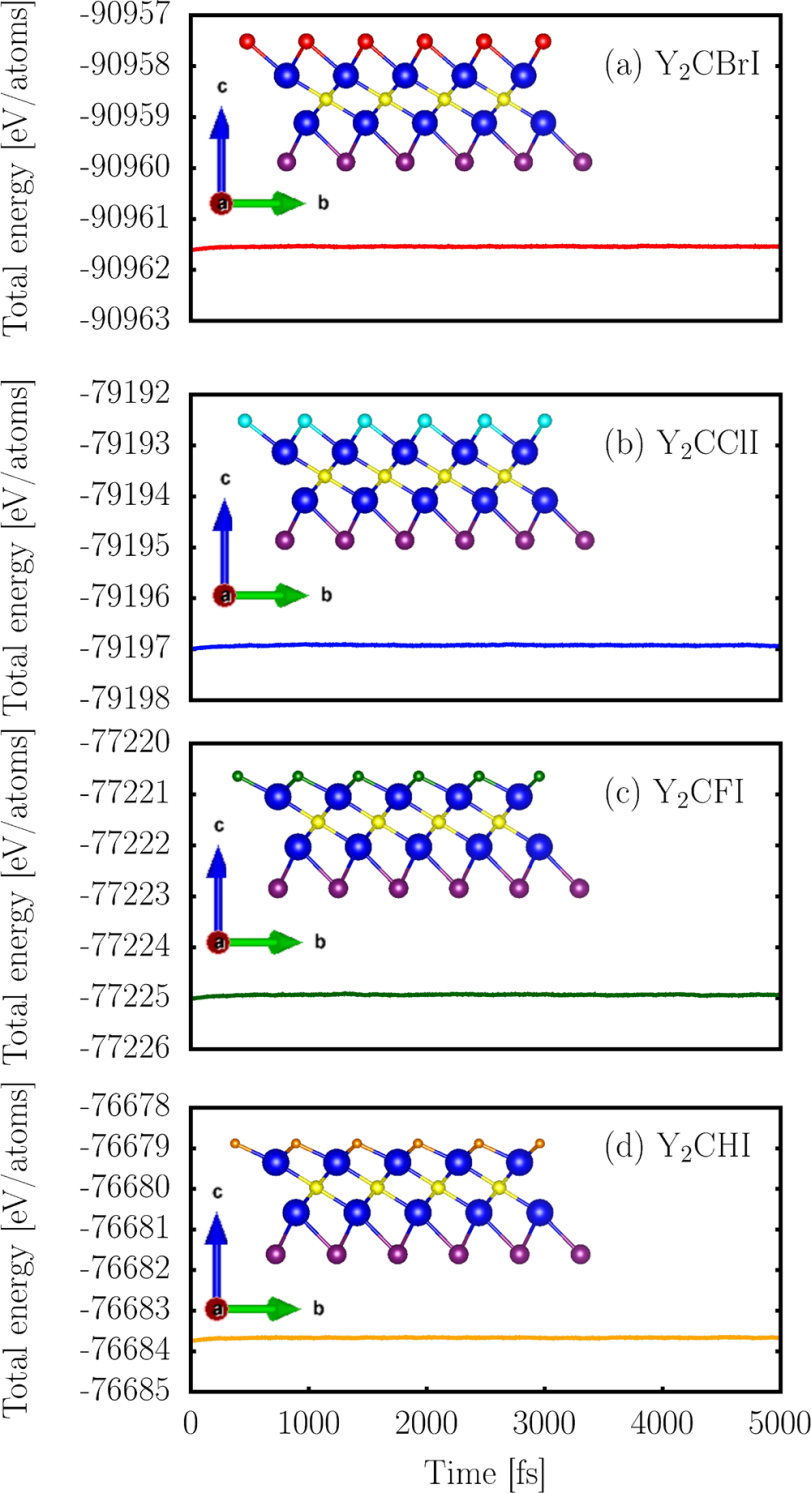
Total energy variation in AIMD simulations of 2D Y_2_CTI monolayers at 300 K.

The second method to confirm the evaluation of the dynamical stability was through phonon plot diagrams of these monolayers, as depicted in Figure 3. A material is considered dynamically stable if its phonon dispersion exhibits no negative frequencies. This condition is satisfied for most materials except for Y_2_CFI, which presents a small negative frequency. However, since these frequencies are obtained through numerical methods and depend on the supercell size used in the calculation, small imaginary frequencies may arise, especially near the Γ point. Such features do not necessarily indicate that the material is dynamically unstable.

**Figure 3:**
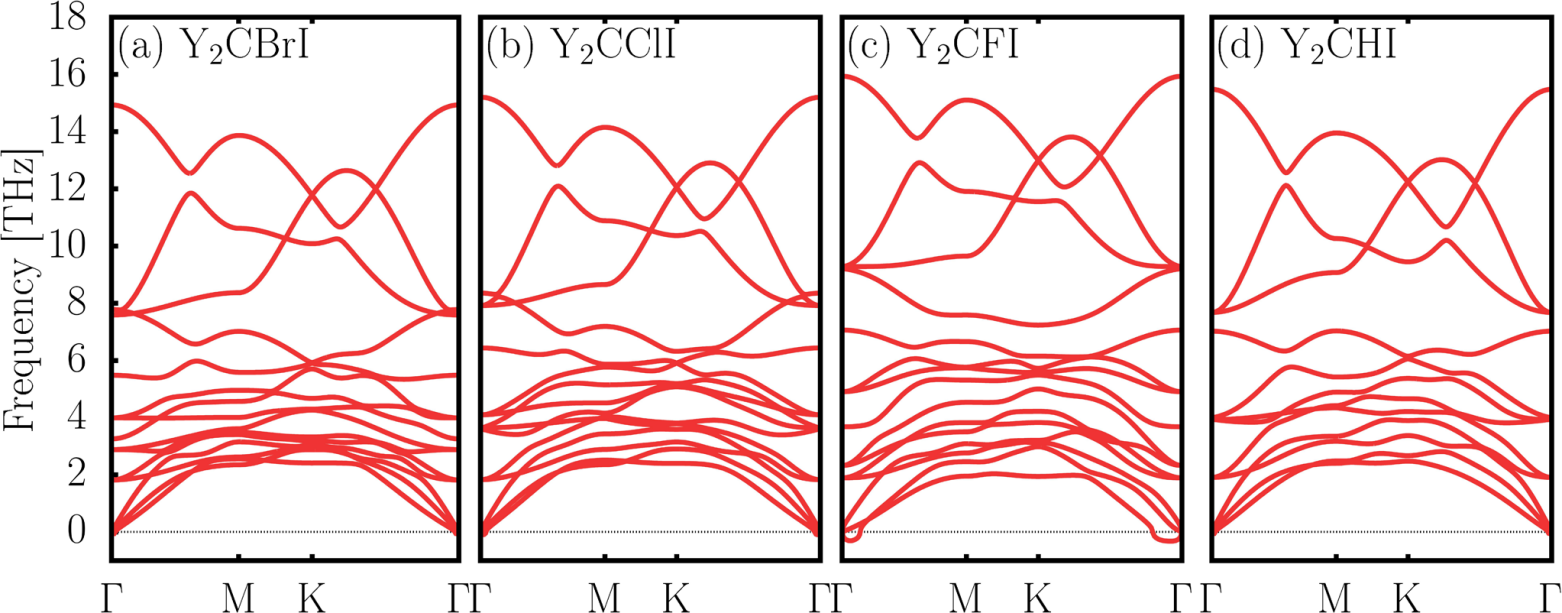
Phonon diagrams of 2D Y_2_CTI monolayers.

Figure 4a shows the calculated curves of Helmholtz free energy *F*(*T*) as a function of the temperature, in the range from 0 to 1000 K. As observed, the transition of the Helmholtz free energy to negative values beyond 300 K indicates that these materials become thermodynamically favorable for synthesis at temperatures exceeding this limit. The *F*(*T*) decreases as the temperature increases, with this being an important characteristic to be considered in studies on the spontaneity of the synthesis of materials. Moreover, Figure 4b shows that at low temperatures in the range from 0 to 300 K, the four materials exhibit similar heat capacity, being proportional to T^3^ as expected by the third law of thermodynamics. However, at high temperatures, Y_2_CHI converged significantly below Y_2_CBrI, Y_2_CClI, and Y_2_CFI, which approached the Dulong–Petit limit^70^ (Cv = 3R) around 600 K, converged near 125 J/K mol. For the Y_2_CHI monolayer, Cv is lower than the other monolayers at any temperature, indicating that the Y_2_CHI monolayer does not heat easily compared to other monolayers.

**Figure 4:**
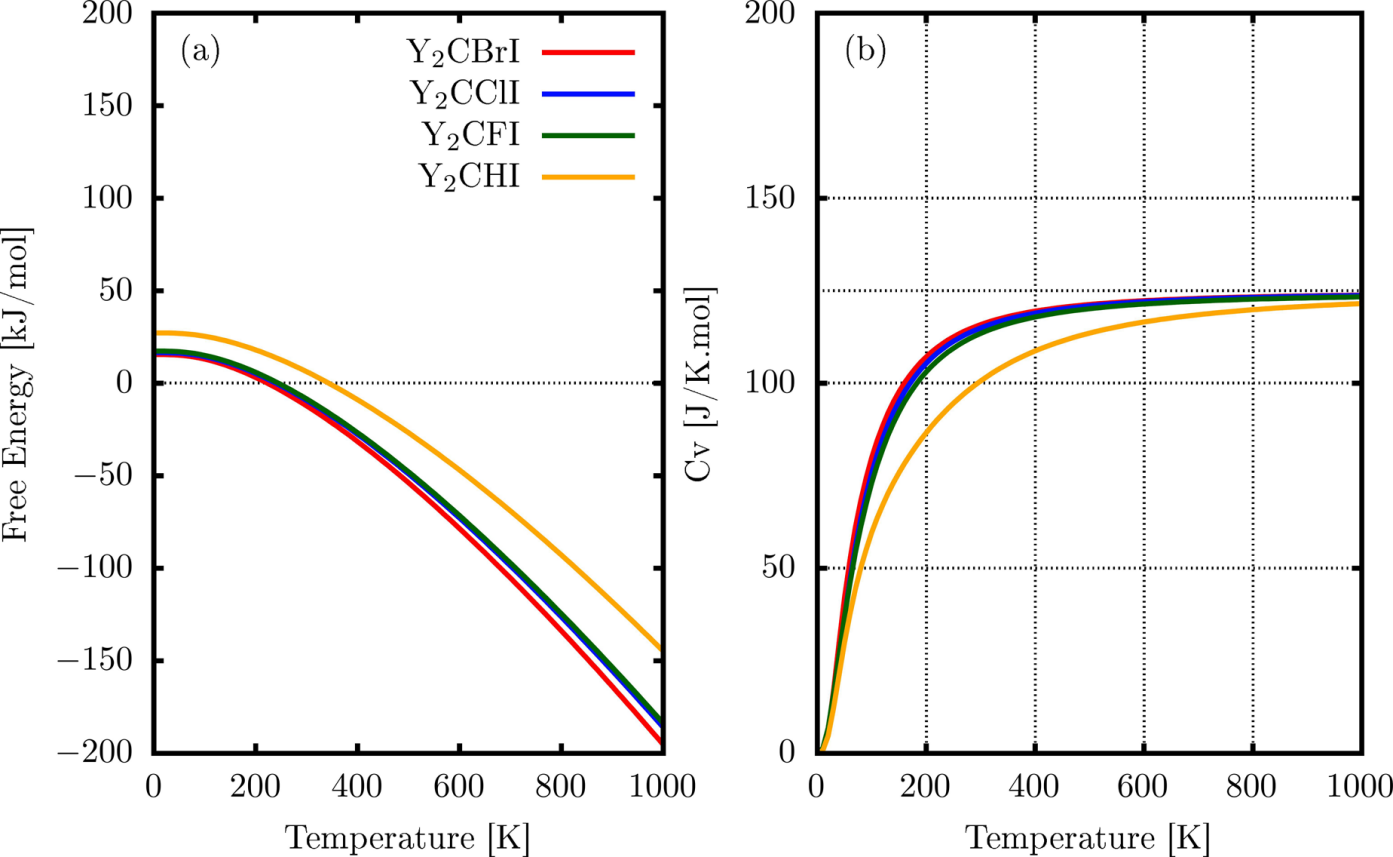
Thermodynamic properties of the 2D Y_2_CTI monolayer: (a) Helmholtz energy *F*(*T*) and (b) heat capacity at a constant volume (Cv) using the PBE method.

Finally, mechanical stability is corroborated by the elastic stiffness coefficients *C_ij_*. For hexagonal systems, there are three elastic constants, stiffness constants, with *C*_11_ and *C*_22_ being necessary, as the third elastic stiffness constant *C*_66_ is obtained from these matrices as

$$C_{ij}=\left(\begin{array}{ccc} C_{11} & C_{21} & 0\\ C_{12} & C_{22} & 0\\ 0 & 0 & (C_{11}-C_{12})/2 \end{array}\right)$$ 3

Table 2 summarizes the calculated elastic constants of the studied 2D Janus MXenes. For hexagonal symmetry, *C*_11_ = *C*_22_ and *C*_12_ = *C*_21_, and *C*_66_ is given as *C*_66_ = (*C*_11_ – *C*_12_)/2. To confirm the mechanical stability, the Born–Huang criteria must be satisfied^71,72^ for a trigonal 2D system, ensuring that *C*_11_ > 0 and *C*_11_^2^ > *C*_12_^2^. It can be seen that all studied monolayers meet the stability criteria, demonstrating the mechanical stability of the Y_2_CTI Janus MXene systems. Among the four monolayers, Y_2_CBrI and Y_2_CClI exhibit the lowest elastic constants, indicating a softer mechanical response likely associated with the larger atomic radii and weaker bonding characteristics of Br and Cl. The Y_2_CHI monolayer follows intermediate values, whereas Y_2_CFI shows the highest elastic constants, reflecting the stronger and more directional bonding induced by the smaller and more electronegative F atom.

**Table 2:** Relaxed Elastic Coefficients, *C_ij_* (N/m), Maximum Young’s Modulus, *Y* (θ) (N/m), and Maximum Poisson’s Ratio ν­(θ) for 2D Y_2_CTI (T = Br, Cl, F, H) Janus MXene Monolayers

system	*C*_11_	*C*_12_	*C*_66_	*Y*_max_	ν_max_
Y_2_CBrI	109.28	24.37	41.87	103.85	0.23
Y_2_CClI	108.91	21.57	43.79	104.81	0.20
Y_2_CFI	128.96	34.22	47.31	119.88	0.27
Y_2_CHI	113.02	19.94	47.36	110.62	0.18

Moreover, the in-plane dependence of Poisson’s and Young’s moduli was evaluated from the elastic constants as follows:

$$Y(\theta )=\frac{C_{11}C_{22}-C_{12}^{2}}{C_{11}s^{4}+C_{22}c^{4}+\left(\frac{C_{11}C_{22}-C_{12}^{2}}{C_{66}}-2C_{12}\right)c^{2}s^{2}}$$ 4

$$\nu (\theta )=\frac{C_{12}(s^{4}+c^{4})-\left(C_{11}+C_{12}-\frac{C_{11}C_{22}-C_{12}^{2}}{C_{66}}\right)c^{2}s^{2}}{C_{11}s^{4}+C_{22}c^{4}+\left(\frac{C_{11}C_{22}-C_{12}^{2}}{C_{66}}-2C_{12}\right)c^{2}s^{2}}$$ 5

where *s* = sin­(θ) and *c* = cos­(θ).

The Y­(θ) and ν­(θ) polar plots show a circular shape, denoting the isotropic mechanical properties of the Y_2_CTI Janus monolayers, as illustrated in Figure 5. The highest values for Young’s modulus (Poisson ratio) are found to be 103.85 (0.23), 104.81 (0.20), 119.88 (0.27), and 110.62 (0.18) N/m corresponding to Y_2_CBrI, Y_2_CClI, Y_2_CFI, and Y_2_CHI, respectively. Our results are consistent with previous theoretical studies.^40,73^

**Figure 5:**
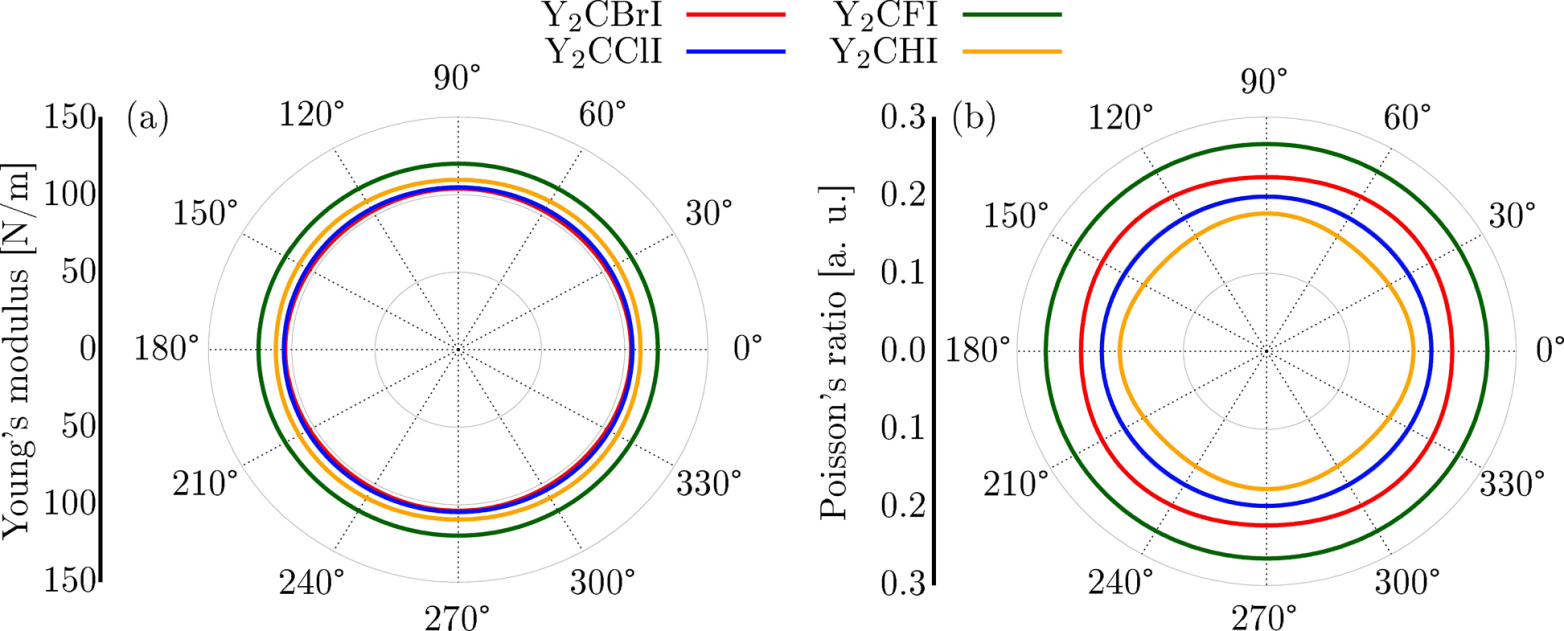
Polar diagram plots for (a) Young’s modulus and (b) Poisson’s ratio of Y_2_CBrI (red color), Y_2_CClI (blue color), Y_2_CFI (green color), and Y_2_CHI (gold color) Janus MXene monolayers.

### 3.2 Electronic Properties

Electronic band structures of Y_2_CTI MXene monolayers were calculated using both the PBE and HSE06 functionals, as shown in Figure 6a–d. Our results show that all monolayers exhibit a semiconducting behavior, characterized by indirect band-gap transitions. Specifically, the valence band maximum (VBM) is located at the Γ point, while the conduction band minimum (CBM) appears at the *M* point. For Y_2_CBrI, Y_2_CClI, Y_2_CFI, and Y_2_CHI, the band-gap energies are at the PBE (HSE06) level of 0.61 (1.33) eV, 0.59 (1.31) eV, 0.58 (1.32) eV, and 0.52 (1.23) eV, respectively. Table 3 presents the band-gap values calculated for each MXene monolayer using the HSE06 hybrid functional.

**Figure 6:**
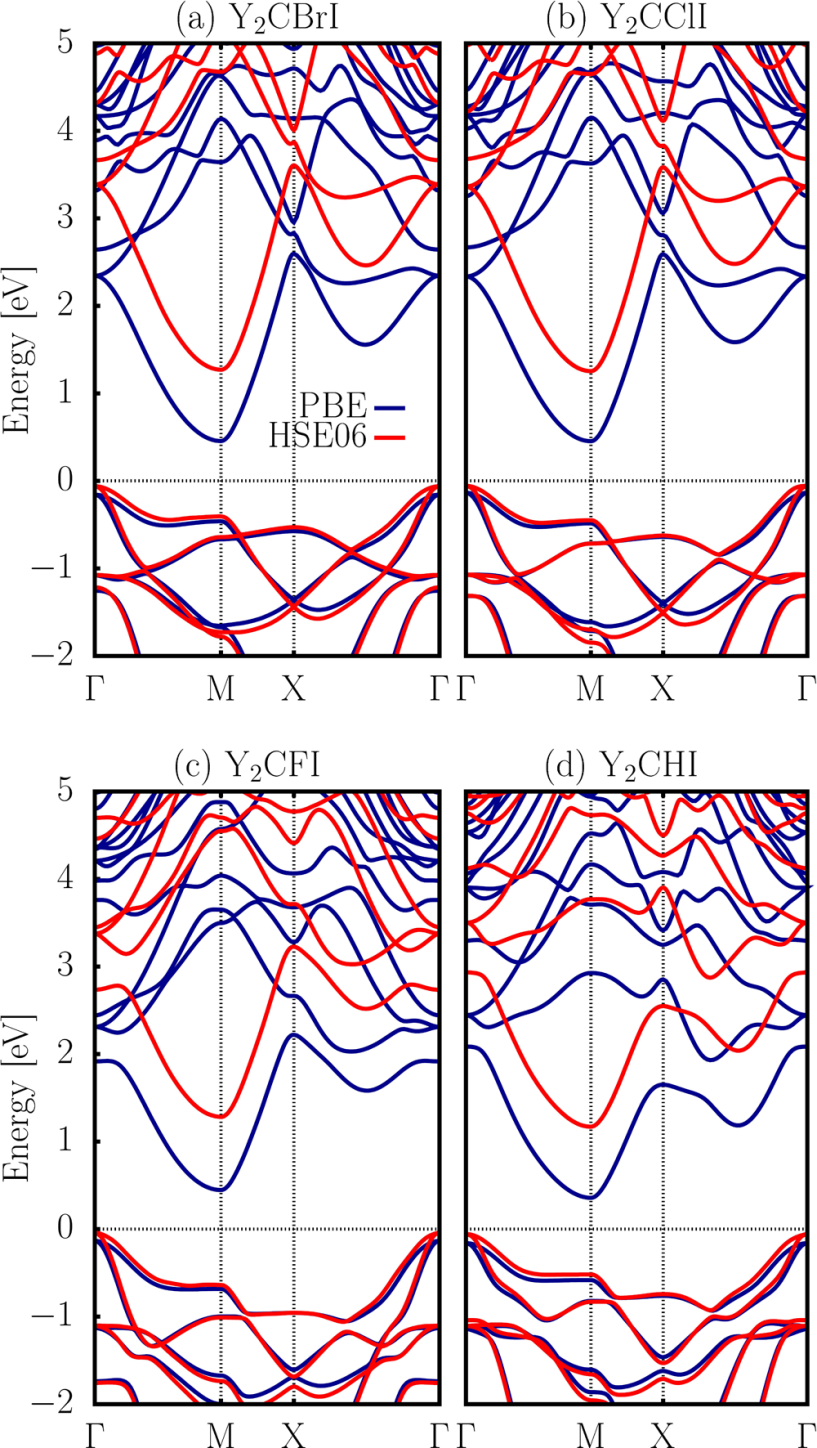
Band structures of 2D Y_2_CBrI, Y_2_CClI, Y_2_CFI, and Y_2_CHI Janus MXene monolayers obtained from PBE and the HSE06 level of functionals. The Fermi level is set at 0 eV.

**Table 3:** Excitonic Properties Obtained with the MLWF-TB + BSE Method (HSE06 Parametrization)^a^

system	E_g_ (eV)	E_g_^d^ (eV)	Ex_gs_ (eV)	Ex_gs_^d^ (eV)	Ex_b_ (meV)
Y_2_CBrI	1.33	1.68	1.10	1.27	228
Y_2_CClI	1.31	1.70	1.09	1.30	218
Y_2_CFI	1.32	1.92	1.12	1.50	200
Y_2_CHI	1.23	1.69	0.99	1.24	325

a Fundamental band gap, E_g_ (eV), direct band gap, E_g_^d^ (eV), exciton ground state, Ex_gs_ (eV), direct exciton ground state, Ex_gs_^d^ (eV), and exciton binding energy, Ex_b_ (meV), calculated as E_g_ – Ex_gs_.

To obtain a deeper understanding, we analyzed the contribution of each atomic orbital to the Y_2_CTI Janus monolayers, as reflected in the projected density of states (PDOS), as shown in Figure 7. The Fermi level is located at 0 eV for the band structure and PDOS. For the Y_2_CBrI monolayer, the valence edge states below 0 to −2 eV are composed mainly of carbon *p* orbitals, followed by a lower contribution of yttrium *d*, bromine *p*, and iodine *p* orbitals, as shown in Figure 7a. However, in the range of −2 to −5 eV, the bromine *p* orbitals are more evident, followed by the iodine *p* orbitals in comparison to the other elements. Meanwhile, the conduction edge states consist of a relatively large amplitude of yttrium *d* orbitals relative to those of the others. Similarly, a trend is shown for Y_2_CClI (see Figure 7b), where the chlorine *p* orbitals exhibit a greater contribution in the range of −3.5 to −5 eV, and the other elements have the same contribution in the valence and conduction edges. However, for Y_2_CFI in Figure 7c, the contribution of PDOS of the fluorine *p* orbital is not evidenced at the valence and conduction energy edges. However, the orbitals of yttrium *d*, carbon *p*, and iodine *p* contribute significantly. Similar results are shown in the Y_2_CHI monolayer, with a minor contribution of hydrogen *s* orbitals, as shown in Figure 7d.

**Figure 7:**
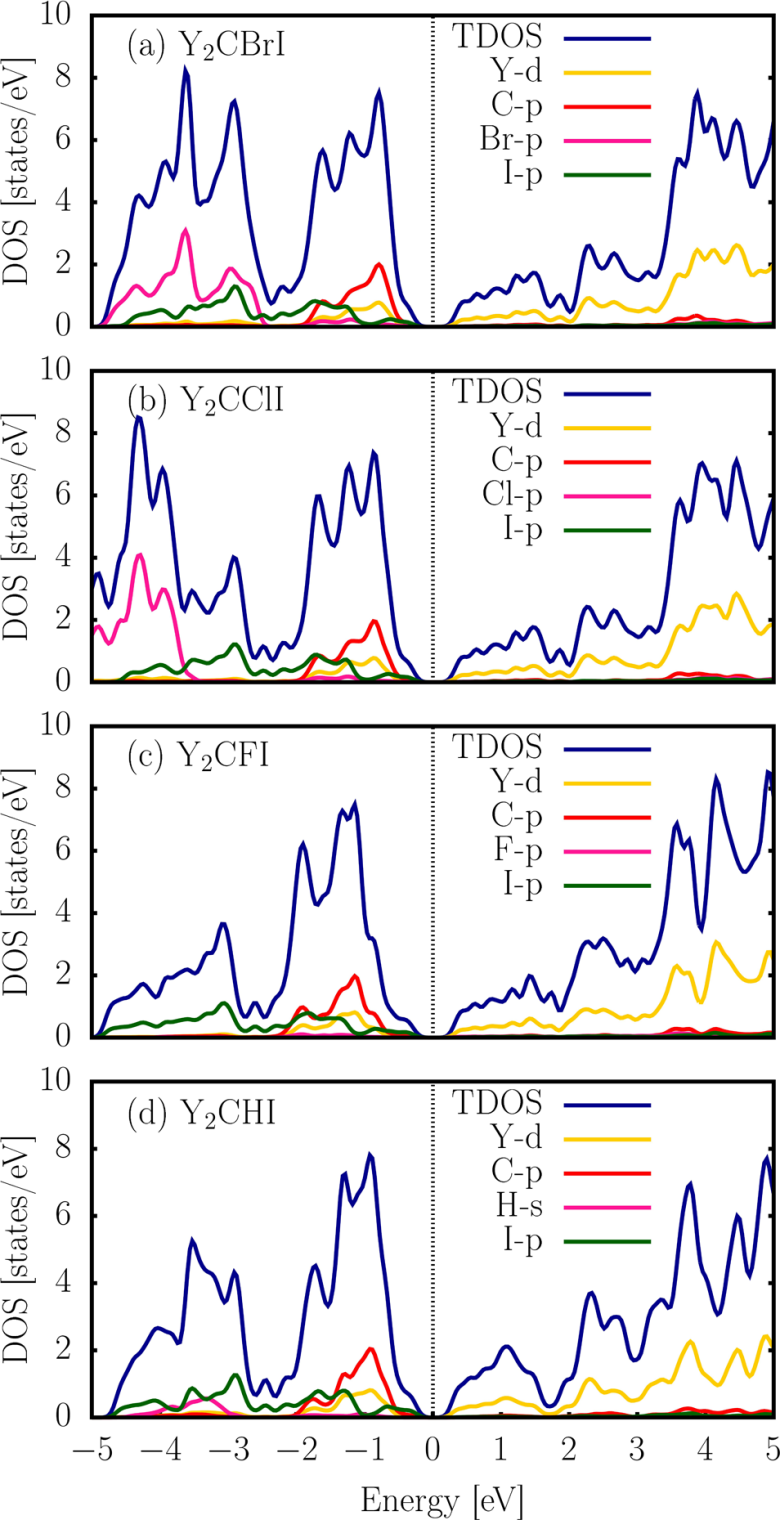
Total and projected electronic density of states (DOS) of 2D Y_2_CBrI, Y_2_CClI, Y_2_CFI, and Y_2_CHI Janus MXene monolayer semiconductors, obtained from the PBE level of theory. The Fermi level is set at 0 eV.

### 3.3 Excitonic Effects and Optical Features

The excitonic electron–hole effects in these Y_2_CTI Janus MXenes were investigated through the Bethe–Salpeter equation (BSE) method. The exciton band structure, shown in Figure 8, demonstrates that the excitonic ground of these Janus monolayers is indirect, independent of its chemical composition, with transitions occurring along the Γ-*M* path for these Janus MXene monolayers. The exciton binding energy (Ex_b_) was calculated considering the difference between the gap (E_g_) and the exciton ground state energy (Ex_gs_), and the values are listed in Table 3.

**Figure 8:**
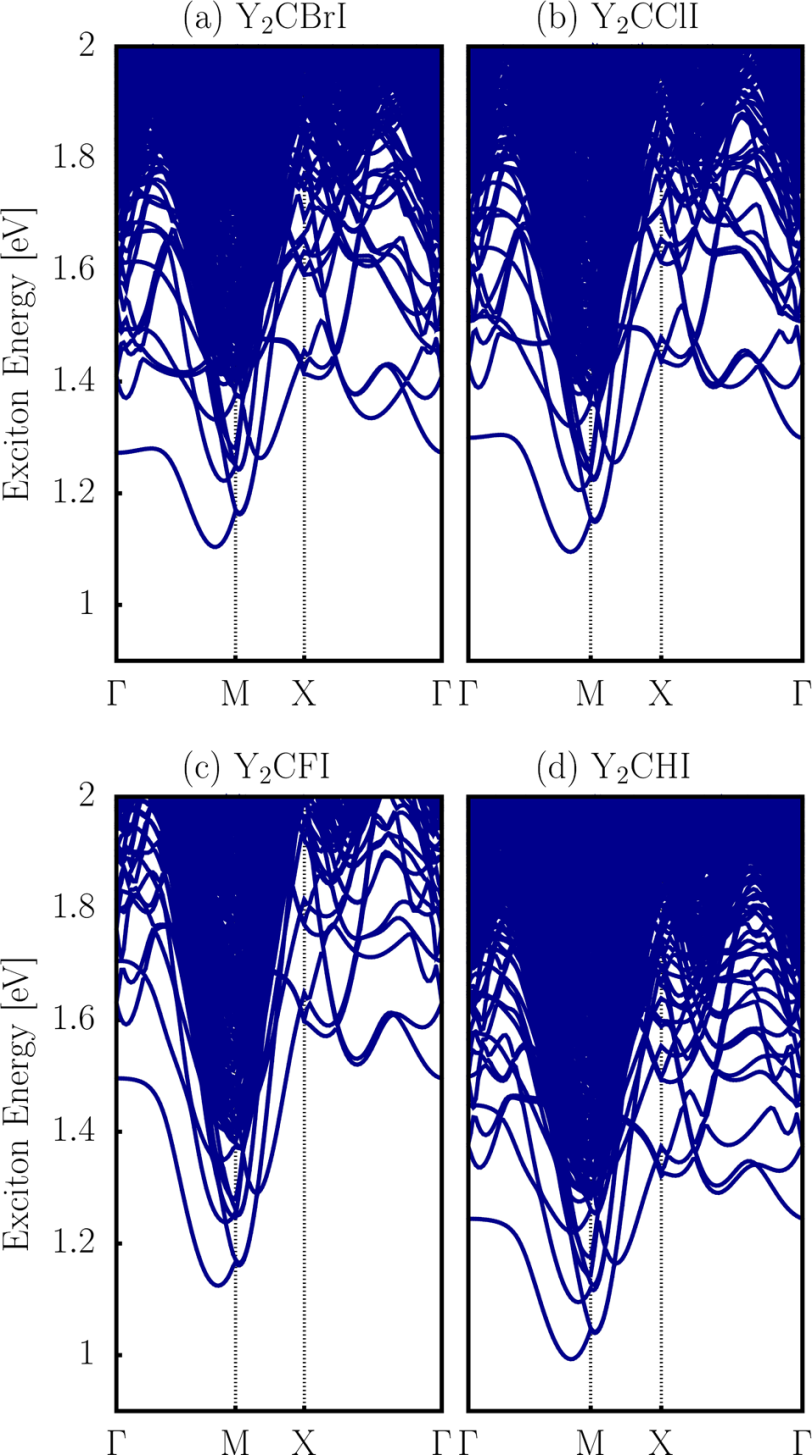
Exciton band structures using the BSE method of 2D (a) Y_2_CBrI, (b) Y_2_CClI, (c) Y_2_CFI, and (d) Y_2_CHI Janus MXene monolayers.

The proposed Janus MXenes exhibit Ex_b_ values ranging from 200 to 325 meV, considering the entire set, with the lowest value for Y_2_CFI and the highest for Y_2_CHI, highlighting the strong quasi-particle effect in these 2D monolayers, which is consistent with typical values for 2D Janus systems.^41,74,75^ In addition, Riis-Jensen et al. showed stable semiconducting MXY Janus monolayers using BSE approximation with band-gap values of 0.7–3.0 eV, having significant exciton binding energies, and very strong light–matter interactions.^76^ These materials also exhibited exciton binding energies from 300 to 1000 meV with a tendency for large exciton binding energies. The Y_2_CBrI and Y_2_CClI showed Ex_b_ values of 228 and 218 meV, respectively. These results indicate a stronger dependence of Ex_b_ on the chemical composition of the monolayer, with the lowest and highest values observed for F–I and H–I asymmetric functionalizations, respectively.

In Figure 9, we present the absorption coefficient calculated using both the BSE and IPA approaches, considering linearly polarized light along the *x* and *y* directions. At the IPA level, the absorption spectra are nearly identical for both polarizations, indicating isotropic light absorption in the IR and visible regions. However, when excitonic effects are included at the BSE level, the absorption features differ between the two polarizations, revealing linear dichroism arising from electron–hole interactions. The optical band gap also varies slightly with polarization, being smaller along the *x* direction (corresponding to the direct exciton ground state). This suggests that polarization filters could be employed to tune the optical band gap via a linear combination of *x*- and *y*-polarized light.

**Figure 9:**
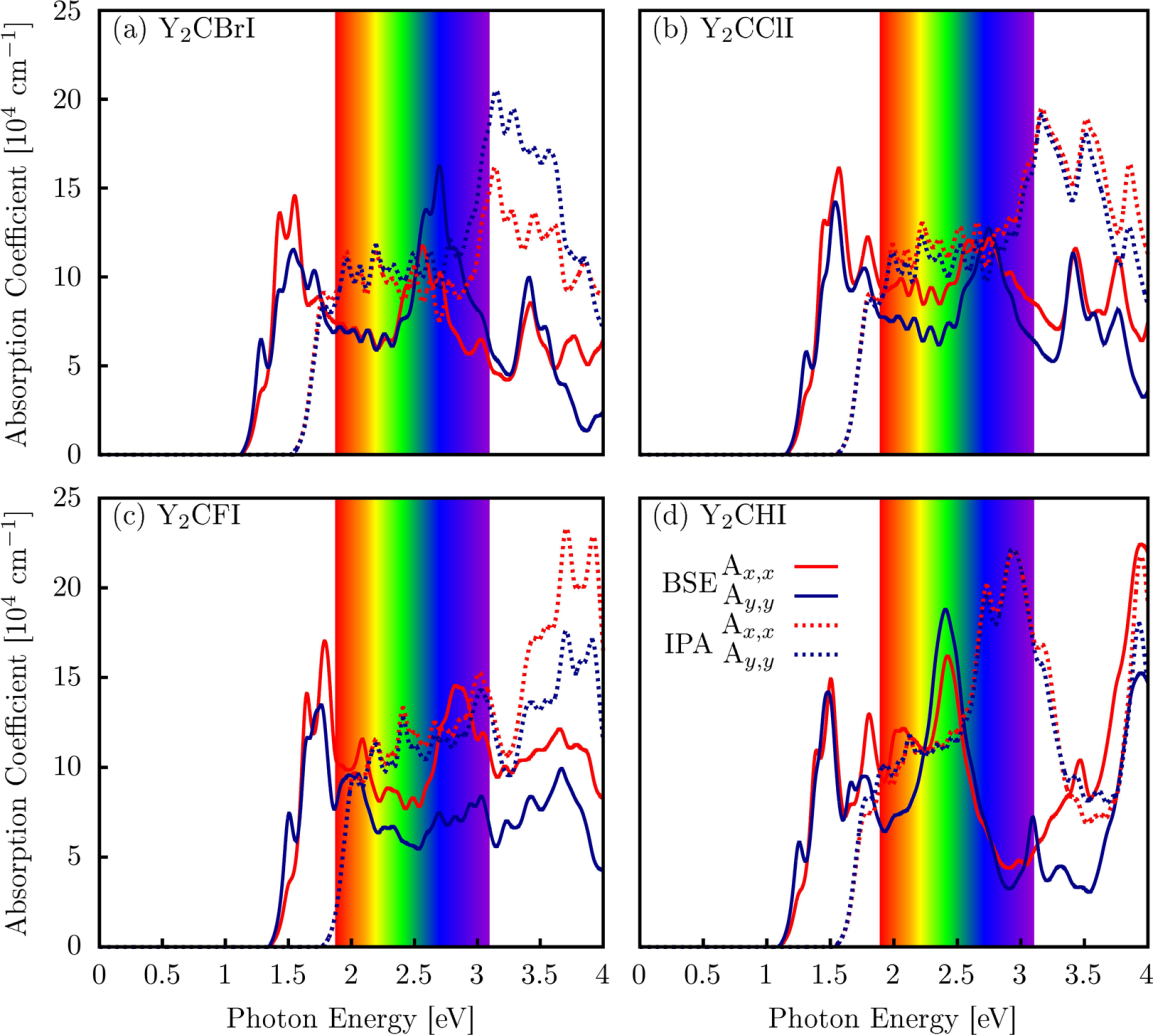
Optical absorption coefficient spectra of (a) Y_2_CBrI, (b) Y_2_CClI, (c) Y_2_CFI, and (d) Y_2_CHI monolayers. The visible spectrum is highlighted in the form of a rainbow. Also, the solid (red and blue) and dashed lines indicate a comparison of BSE and IPA absorption spectra, respectively.

The first absorption peaks for the Y_2_CBrI using the BSE level are in the IR region from 1.3 to 1.85 eV, showing a significant absorption coefficient (15 × 10^4^ cm^–1^) in the *x* light polarization, as illustrated in Figure 9a. Also, the main absorption coefficient edge in the visible region at about 2.7 eV is shown with approximately 16.5 × 10^4^ cm^–1^ in the *y* light polarization. In the ultraviolet (UV) region, the absorption coefficient is slightly reduced. However, at the IPA level, the main absorption peaks are in the UV region up to a value of 20 × 10^4^ cm^–1^. In the other structures, the band-gap variation caused considerable changes in the absorption coefficients when the systems are different (see Figure 9b,c for Y_2_CClI, Y_2_CFI, and Y_2_CHI). All MXene monolayer systems still show absorption peaks in the IR, visible, and UV regions with absorption coefficients around 10^4^ cm^–1^, but the absorption spectra are red-shifted and the first absorption peaks are declined using the BSE level (quasi-particle effect). The fundamental band gap (E_g_) and direct band gap (E_g_^d^) are located in the IR and visible regions with a superior absorption efficiency, making it a strong candidate for optoelectronic applications. Also, these results exhibit an excellent absorption response in the IR, visible, and UV regions, as illustrated in Figure 9a–d, suggesting potential applications for UV sensor detectors, solar cells, and IR sensor technologies.

### 3.4 Interaction with Light: PCE

The interaction of the chosen Y_2_CTI monolayers with light was investigated for solar harvesting efficiency of the proposed MXene Janus monolayers through the analysis of the PCE across three different theoretical models: the PCE obtained through SLME (PCE^SLME^), the maximum theoretical efficiency under SLME (PCE_max_^SLME^), which assumes 100% photon absorption starting from the band gap,^44^ and the Shockley–Queisser efficiency limit (PCE^SQ^), which considers only radiative recombination losses and assumes ideal semiconductor behavior.^43,77^ The calculated PCE for each of these levels is shown in Table 4.

**Table 4:** Maximum PCE at the IPA and BSE Levels and PCE obtained by SLME (PCE^SLME^) (%)^a^

	IPA			BSE		
system	PCE^SLME^	PCE_max_^SLME^	PCE^SQ^	PCE^SLME^	PCE_max_^SLME^	PCE^SQ^
Y_2_CBrI	0.82	20.95	28.73	0.88	25.92	31.88
Y_2_CClI	0.83	19.74	28.26	0.95	24.99	32.07
Y_2_CFI	0.57	14.48	24.08	0.72	20.75	31.28
Y_2_CHI	0.75	18.29	28.29	0.80	22.81	31.98

a PCE obtained by SLME considering 100% of the photon absorbance starting from the band gap (PCE_max_^SLME^) (%), and PCE obtained in the Shockley–Queisser limit considering the band gap (PCE^SQ^)­(%), considering the solar device at room temperature *T* = 300 K.

To calculate the PCE of these 2D Janus MXene materials, we accounted for direct and indirect transition band gaps. Furthermore, we consider the ground-state excitonic transitions shown in Figure 8. All of these parameters are listed in Table 3. With this information, the upper limit PCE is calculated using the following expression:^3,78^

$$\mathrm{PCE}=\frac{J_{\mathrm{SC}}V_{\mathrm{OC}}}{P_{\mathrm{SOLAR}}}$$ 6

where *V*_OC_ indicates the maximum open circuit voltage, *J*_SC_ is the short circuit current, and *P*_SOLAR_ represents the total incident solar power per unit area, defined as

$$J_{\mathrm{SC}}=\int_{E_{g}^{d}}^{\infty }\frac{P(E)}{E}dE$$ 7

and

$$P_{\mathrm{SOLAR}}=\int_{0}^{\infty }P(E)dE$$ 8

Here, E_g_^d^ corresponds to the direct electronic band gap of the material. In contrast, *P*(*E*) represents the Air Mass global solar spectral irradiance (AM1.5G^66^), with a solar zenith angle of 48.2°, the reference spectrum for standard photovoltaic performance under typical nonconcentrated sunlight conditions. To incorporate quasi-particle effects in the PCE calculations, we substituted the electronic band gap with the optical band gap of the studied monolayer, obtained from the direct excitonic ground state Ex_gs_^d^. This strategy allows for a more detailed assessment of the photovoltaic capabilities of these materials, as electron–hole interactions have a significant influence on absorption and charge carrier dynamics in two-dimensional semiconductors.

Initially, we examine PCE^SLME^, considering that the atomic-scale layer thickness characteristic of 2D materials restricts light absorption. The investigated Janus materials have values below 1.00%. The PCE^SLME^ calculated at the BSE level falls in the range between 0.72 and 0.95%; however, at the IPA level, we have values between 0.57 and 0.83%, revealing the beneficial impact of Janus structures on light conversion efficiency, calculated at the BSE level.

On the other hand, Table 4 also presents PCE_max_^SLME^ considering maximum light absorption that can be researched through device design.^79^ The BSE approach yields significantly enhanced efficiency values for all monolayers, ranging from 20.75 to 25.92%, whereas the IPA approach predicts comparatively lower PCE_max_^SLME^ values in the range of 14.48–20.95%. Notably, the Y_2_CBrI Janus MXene exhibits the highest predicted efficiency (25.92%), surpassing that of the other Janus configurations.

Table 4 further shows the PCE^SQ^ values, which are correlated with the electronic band gap of the material. As expected, the PCE^SQ^ results are higher than those obtained for PCE_max_^SLME^, with Y_2_CBrI having 31.88%, Y_2_CClI 32.07% (highest value), and the Y_2_CFI 31.58% and Y_2_CHI 31.98% at the BSE level. A similar trend is observed for the IPA level. It is important to note that in the IPA approximation, the PCE^SQ^ values range between 24.08 and 28.73%. The discrepancy between these values and those obtained using the SLME method (PCE_max_^SLME^) is attributed to the recombination fraction (fr), which was obtained through the difference between Ex_gs_^d^ (E_g_^d^) and Ex_gs_ (E_g_) at the BSE and IPA levels, respectively. Since this difference is significantly larger in BSE calculations, the efficiency values predicted using this method tend to be lower than those derived from the IPA approximation.

In our previous publications, heterojunctions of TMD MoTe_2_/MoSe_2_, MoSe_2_/WS_2_, and MoTe_2_/WS_2_, MoSe_2_/WS_2_ showed a PCE around 20%.^3^ Also, 2D carbon allotropes exhibited a wide range of PCE values, from 7% to 30%, assuming all absorption of incident light.^10^ Additionally, some scandium- and yttrium-based MXene monolayers demonstrated solar harvesting efficiencies exceeding 30% with the inclusion of quasi-particle effects.^35^ In our work, Janus Y_2_CTI monolayers show the highest efficiency in the conversion of solar energy around 32% and demonstrate the theoretical potential of 2D MXenes in the Janus form as photoabsorbers for solar energy harvesting applications. Our predicted PCE efficiency for solar cells, which approaches the maximum Shockley–Queisser limit, represents a significant advancement in the field of optoelectronics.

## 4 Conclusions

Therefore, we theoretically predicted the optoelectronic properties of Janus MXene monolayers based on first-principles calculations and a semiempirical approach based on the maximally localized Wannier function tight-binding (MLWF-TB) model. Theoretical prediction results indicate that 2D Janus MXenes exhibit excellent optical properties for photovoltaic device applications, achieving optimal PCE between 31 and 32%. Exciton binding energies, which ranged from 200 to 350 meV, induced a red-shift in the optical band gap, significantly impacting the solar harvesting potential. Furthermore, our results present the electronic band gaps of semiconductors, calculated using the HSE06 method, ranging from 1.23 to 1.33 eV with indirect band gaps. Additionally, our results clarify the shortcomings of previous theoretical optical spectra and provide the opportunity to design tunable optoelectronic components for photovoltaic devices.
